# siRNAs Induce Efficient RNAi Response in *Bombyx mori* Embryos

**DOI:** 10.1371/journal.pone.0025469

**Published:** 2011-09-30

**Authors:** Junichi Yamaguchi, Takayuki Mizoguchi, Haruhiko Fujiwara

**Affiliations:** Department of Integrated Biosciences Graduate School of Frontier Sciences, The University of Tokyo, Kashiwa, Chiba, Japan; German Cancer Research Center (DKFZ) and Heidelberg University, Germany

## Abstract

Short interference RNA (siRNA) is widely used in mammalian cells. In insects, however, reports concerning the suitablility of siRNA *in vivo* is very limited compared with that of long dsRNA, which is thought to be more effective. There is insufficient information on the essential rules of siRNA design in insects, as very few siRNAs have been tested in this context. To establish an effective method of gene silencing using siRNA *in vivo* in insects, we determined the effects of siRNA on seven target genes. We designed siRNAs according to a new guideline and injected them into eggs of *Bombyx mori*. At the mRNA level, the expression of most of these genes was successfully silenced, down to less than half the constitutive level, which in some cases led to the development of distinctive phenotypes. In addition, we observed stronger effect of siRNA both on the mRNA level and the phenotype than that of long dsRNA under comparable conditions. These results indicate that direct injection of siRNA is an effective reverse-genetics tool for the analysis of embryogenesis *in vivo* in insects.

## Introduction

RNA interference (RNAi) is a powerful tool for knocking down the expression of target genes. The phenomenon, in which a double-stranded RNA (dsRNA) is critical for the suppression of the target gene, was first discovered in *Caenorhabditis elegans* [1] and was described subsequently in insects [2] and in other animals [3]. The mechanism is conserved in eukaryotes: the dsRNA introduced into cells is processed by Dicer, a member of the ribonuclease III family, into short interference RNAs (siRNAs) of 21–23 nt, which then guide the multicomponent nuclease complex termed RNA-induced silencing complex to the target mRNA, for degradation [4].

The use of RNAi in mammalian cells was limited at first, as the introduction of long dsRNAs into the cytoplasm of cells frequently triggers a fatal interferon response [5]. However, once it became clear that siRNAs can induce gene silencing without activating the interferon response [6], their introduction became a promising approach for RNAi in mammalian cells.

In contrast to the mammalian case, long dsRNAs can mediate RNAi in insects without inducing a cytotoxic response; therefore, direct injection of long dsRNAs is recognized as a first choice for RNAi experiments in this context. However, as synthetic siRNAs are becoming cost effective and the sequences of many insect genes are becoming available from databases, direct injection of synthetic siRNAs should be desirable not only for basic-research purposes but also for high-throughput screening of the function of unknown genes.

Although we recognize the importance of the use of siRNA in insects, the knowledge available on the effect of siRNA *in vivo* in insects is limited, as there are very few examples of siRNAs that have been tested in insects. We do not know whether siRNAs designed using a commercial guideline fitted for mammalian genes are also effective in insects. In mammalian cells, it is reported that variation of only 1 or 2 bp in the target sequence has a considerable effect on the activity of RNAi [7], [8]. Thus, to establish a reliable guideline for the design of effective siRNAs in insects, we need to collect many examples of siRNA tested using comparable conditions.

Recent studies revealed the success of RNAi using long dsRNAs during embryogenesis in the silkworm *Bombyx mori*, a Lepidoptera model insect, whereas larval RNAi (direct injection of synthetic dsRNA into the larval body or organs) was seriously limited [9]. Here, we focused on four genes, *ultrabithorax* (*Ubx*), *wnt1/wingless* (*wnt1*), *odd-skipped* (*odd*), and *tyrosine hydroxylase* (*TH*), to elucidate the effect of the direct injection into eggs of siRNAs designed using a novel guideline based on several rules specific for mammalian cells. We tested another three genes and confirmed that the guidelines devised were adaptable to *in vivo* experiments in *B. mori* and that the some siRNAs could induce stronger RNAi effect on both phenotypes and mRNA levels than that of long dsRNAs.

## Results and Discussion

### Our guideline for the design of siRNAs

**Figure 1:**
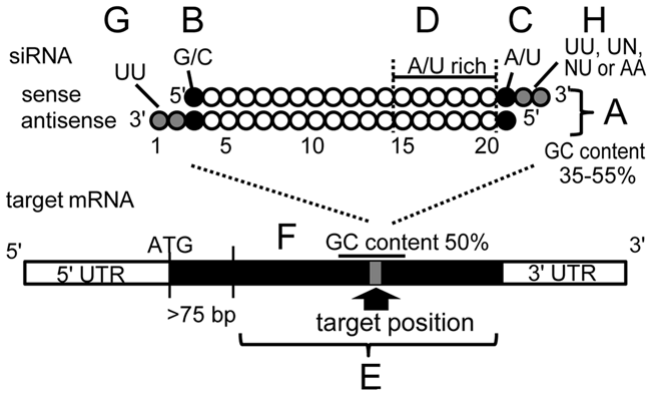
Schematic illustration of the design of effective siRNAs. All residues of siRNA (filled or open circles) corresponded to those of the target mRNA sequences, except for the 3′ overhangs of the sense strand. In addition, the following conditions were preferred: (A) siRNAs with a GC content that ranged between 35% and 55%; (B) the 5′-sense end of siRNAs was G or C [7]; (C) the 5′-antisense end of siRNAs was A or U [7]; (D) the target position selected was located 75 bp downstream of the initiation codon (AUG), to avoid binding sites for translational factors [13]; (E) a GC content of 50% was preferred in the 12 bp sequences flanking the target position [14, Japan Bio Services Co.,LTD.(JAPAN) unpublished data]; (F) presence more than 3 nt of A or U residues in a 7 nt sequence at the 5′ end of the antisense strand of siRNA [7]; (G) the 2 nt overhangs of the antisense strand at the 3′ end were UU [15]; and (H) the 2 nt overhangs of the sense strand at the 3′ end were UU, UN, NU, or AA [15, Japan Bio Services Co.,LTD.(JAPAN) unpublished data].

Although there are a few examples of siRNA-mediated gene targeting [10]–[12], the effective design of synthetic siRNAs remains unclear in insects. It was reported previously that almost all of the usable sequences in mammalian cells are also effective in insect (*Drosophila*) cells, with some exceptions [7]. Thus, to design siRNAs in insects, we employed four criteria (A to D in Fig. 1), gathered mainly from the report of Ui-Tei *et al.*, and we added another four criteria (E to H in Fig. 1) to ensure the effectiveness of the siRNAs in this model.

First, we followed criteria A to D, which pertain to information about the sequence of the siRNA itself. The target mRNA sequence was checked as follows: (A) GC content of the siRNA was 35–55%; (B and C) the first base of the sense and antisense strand was G/C and A/U, respectively; and (D) the seven bases of the 5′ end of the antisense strand were AU rich (more than three A/U bases). We selected preferentially candidate siRNA sequences that matched all these criteria.

As other reports suggest that the surrounding sequences of siRNA in the target mRNA are also important for the effectiveness of the siRNAs, we added the following two criteria to our guideline: (E) the target sequence selected should be located more than 75 bp downstream of the initiation codon (AUG) [13]; and (F) a GC content of 50% in the 12 bp of sequence flanking the target position should be preferred [14, Japan Bio Services Co.,LTD.(JAPAN) unpublished data]. We also added the following two criteria: (G) the 2 nt overhang of the antisense strand at the 3′ end should be UU (Fig. 1 G) [15]; and (H) the 2 nt overhang of the sense strand at the 3′ end should be UU, UN, NU, or AA (Fig. 1 H) [15] (Japan Bio Services Co.,LTD.(JAPAN) unpublished data). Among the sequences that met criteria A to D, we selected those that also met criterion E. Thereafter, among these, we selected the two sequences (siRNAs A and B) that fitted criteria F to H best and evaluated for specificity of the sequences using BLASTN search of the silkworm EST databases (CYBERGATE, http://150.26.71.213/cgi-bin/main_MX).

### Direct injection of *Ubx*-siRNA into eggs of *B. mori* yielded clear phenotypic effects

The *Hox* gene *Ubx* was chosen as a target gene to evaluate the effect of siRNA injection into eggs of *B. mori*. Reportedly, knockdown of *Ubx* using long dsRNA injected into eggs results in the development of an additional pair of thoracic leg-like protuberances in the A1 segment [16], [17]. This is explained by the conserved function of suppression of development of abdominal appendages, and this phenotype is easily distinguishable from the normal phenotype. We designed two siRNAs (*Ubx*-siRNA-a and *Ubx*-siRNA-b) in accordance with the guideline described in Fig. 1 and injected a mixture containing 25 µM of each siRNA (50 µM in total) into eggs of *B. mori*. As mentioned previously [17], direct injection of reagents into eggs of *B. mori* results in some abnormalities, including developmental arrest, which are presumably caused by the injection process itself. Therefore, we injected the buffer alone as a control experiment. We confirmed that the hatch rate of all injected eggs was 60% and that no significant abnormalities were observed in the control developed embryos (Table 1).

**Table 1:** Effect of siRNAs on embryos of *B. mori*.

target gene	siRNA	µM	hatch rate*	phenotype**	mRNA level***
buffer (control)	-	0	60% (165/273)	-	-
*Bm_Ubx*	a+b	50	7% (15/197)	90% (123/136)	-
(day1)****	a+b	50	41% (57/140)	13% (3/23)	-
(day2)****	a+b	50	50% (74/148)	0% (0/48)	-
*Bm_wnt1*	a+b	50	0% (0/51)	97% (37/38)	-
*Bm_Odd*	a+b	50	3% (2/64)	33% (11/33)	-
	A	50	6% (5/81)	79% (38/48)	-
	b	50	13% (4/30)	5% (1/22)	-
8846 (unknown)	a+b	50	76% (71/94)	-	0.11
*Bm_ago3*	a+b	50	55% (52/94)	-	0.47
*Bm_spz3*	a+b	50	48% (45/94)	-	0.82
	c+d	50	56% (27/48)	-	0.46
*Bm_TH*	a+b	100	-	>90%	0.07
	a+b	50	27% (13/47)	>90%	0.11
	a+b	50	-	>90%	0.12
	a+b	25	-	>90%	0.56

*The number of hatched embryos/the number of injected eggs.

**Ratio of embryos with each characteristic phenotype as shown in Fig. 3 in developed embryos.

***Efficiency of injected siRNAs estimated by real-time RT-PCR. The mRNA level was measured in both siRNAs and buffer injected embryos and calculated the ratio. All cDNAs were synthesized from 9 days embryos.

****Ubx-siRNA injection 1 or 2 day after oviposition.

**Figure 2:**
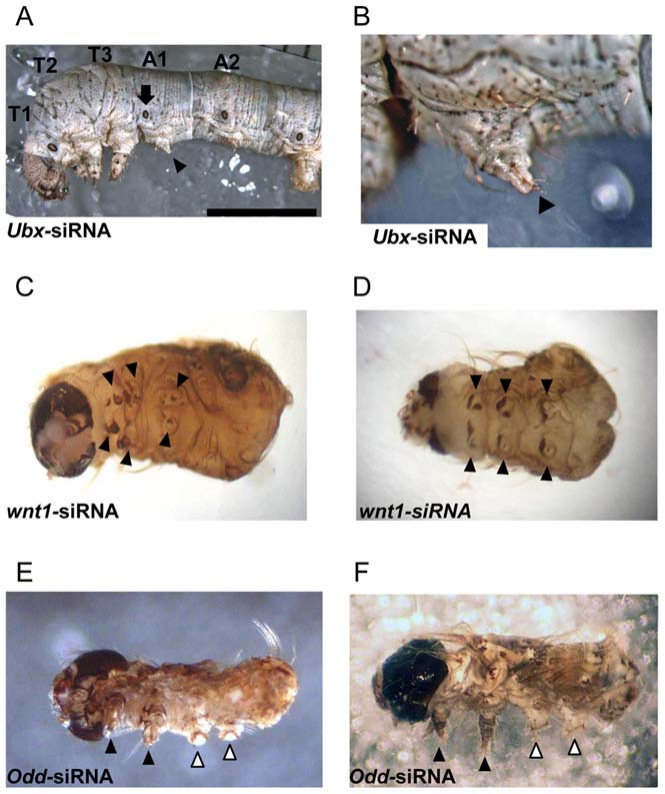
Target-specific effects on phenotype. (A) A lateral view of a larva (fifth stage) injected with the *Ubx*-siRNA 5 h after oviposition. Scale bar, 0.5 mm. An additional pair of legs was observed in A1 (denoted by the arrowhead). The arrow denotes the spiracle that remained in A1, which is the mark of the unique role of *Bm-Ubx* (which is restricted to the ventral part) [16]. (B) Enlargement of the additional leg at the A1 segment of the larva shown in (A). Claw-like structures were observed on the tip of the additional legs (arrowhead), as reported previously [16]. (C) A lateroventral view of the *wnt1*-siRNA injected larva. Filled arrowheads denote the legs. Abdominal prolegs disappeared, corresponding to the absence of abdominal segments. Other individual larvae injected with the *wnt1*-siRNA are shown in (D) and in Fig. S1 B. (E) and (F) Lateroventral and lateral views of *odd*-siRNA-injected larvae. Two pairs of legs (black arrowheads) and prolegs (white arrowheads) were observed. The numbers of legs and prolegs were abnormal compared with those of untreated larvae.

Most embryos injected with *Ubx*-siRNA did not hatch (only 7% hatched), and a similar phenotype exhibiting additional leg-like structures in the A1 segment was observed in 90% of developed embryos (Table 1, Fig. S1). Unexpectedly, a few individuals (less than 10%) with the same phenotype were found in hatched larvae and survived to the last larval stage (fifth stage, Fig. 2 A and B). The additional pair of leg-like protuberances in the A1 segment contained setae, shallow grooves, and claw-like structures at the tips (Fig. 2 B, arrowhead), suggesting the successful silencing of the function of *Ubx*. Furthermore, we confirmed that the spiracles remained in the A1 segment (Fig. 2 A, arrow), which is considered to be a specific phenotype associated with the silencing of *Ubx* function in *B. mori*.

To compare the efficiency of siRNAs with that of dsRNAs, we made long dsRNA of *Ubx* (500 bp long, Table S3) and injected it into the eggs using the same system for siRNA. The injection of long dsRNA of *Ubx* at a concentration of 2.4 µg/µL induced the same phenotype (Fig. S2 B) as seen in the *Ubx* siRNA injection. The phenotypic effect was, however, observed only in 14% of the embryo injected with long dsRNA, which is much lower than 90% in the siRNA injection. The hatch rate in the injection of long dsRNA of *Ubx* was higher (80%) than that of the siRNA injection (7%). A previous study reported that *Ubx* RNAi shows lower hatch rate [16], and thus the above results suggest that siRNA induce RNAi stronger than long dsRNA in this case.

### siRNAs for five additional target genes yielded phenotypic effects or caused reduction of mRNA levels

To demonstrate the target specificity of the injected siRNAs, other target genes (*wnt1* and *odd*) were investigated in the same way as *Ubx*. Although these two genes have not previously been functionally analyzed in *B. mori*, their functions are conserved among a broad range of species [18], [19]. Therefore, the effect of direct injection of siRNAs can be determined by observing the resulting embryonic phenotypes. We injected siRNAs into eggs and observed the developing embryos just before hatching.

*Wnt1* is one of the ligands of WNT signaling, which plays a crucial role in embryonic posterior segmentation [20]. In *wnt1*-siRNA embryos, unlike what was observed for the *Ubx*-siRNA, the abdominal segments were truncated or fused (97% of developed embryos), and three pairs of immature thoracic legs, which characterize the thoracic segments, were observed (Fig. 2 C and D). This suggests that the depletion of *wnt1* by the injection of *wnt1*-siRNA reduces WNT signaling, which is necessary for posterior segmentation in the embryos of *B. mori*.

In the case of *odd*-siRNA injection, 33% of developed embryos exhibited a characteristic phenotype with abnormalities in the number of thoracic legs and abdominal prolegs, which was distinguishable from that observed for the *Ubx*-siRNA and *wnt1*-siRNA. *odd* was identified as a *Drosophila* pair-rule gene that yields a mutant phenotype characterized by pattern deletions of the anterior portions of the odd-numbered segments, which are partially replaced by mirror-image duplications of adjacent elements [21]. As shown in Fig. 2 E and F, some individuals had two pairs of mature legs and prolegs, whereas a wild-type embryo had three pairs of legs and four pairs of prolegs in the T1–T3 and A3–A6 segments, respectively. This phenotype, which represents a deletion of the T2, A3, and A5 segments suggests that the function of *odd* in segmentation in *B. mori* is similar to that observed in *D. melanogaster*.

To compare the efficiency of siRNA-mediated gene knock-down in *B. mori* with that induced by longer dsRNA fragments, we generated long dsRNAs for *wnt1* and *odd* and injected them at a concentration of 2.4 µg/µL in the same way for siRNAs. As shown in Fig. S2 C and D, similar phenotypes as with the siRNA injection were observed in each injection of long dsRNA. It is of interest that these phenotypes by long dsRNA were observed at lower rates than those by siRNA (*wnt1*, long dsRNA 9% (4/46 individuals), *siRNA* 97%; *odd*, long dsRNA 2% (1/50 individuals), siRNA 33%), suggesting that siRNA is more effective for RNAi than long dsRNA, as shown in *Ubx*.

In contrast to the results obtained for the two genes described above, three siRNAs targeting *8846*, *Bm_ago3*, and *Bm_spz3*, whose functions in embryogenesis are not known, showed no visible effects on the morphology and hatch rate of injected embryos (Table 1). However, we observed a reduction in the mRNA levels of *8846*, *Bm_ago3*, and *Bm_spz3* 9 days after hatching (11%, 47%, and 82%, respectively, Table 1). Although *spz3*-siRNA-a and -b yielded a small reduction in mRNA levels, the additional pair of siRNAs for this target (*spz3*-siRNA-c and -d) yielded a clearer reduction of mRNA levels (46%), indicating that the latter two are more suitable for RNAi (see below). These reductions of mRNA levels in the siRNA injection were also confirmed to be stronger than those of long dsRNA at a concentration 2.4 µg/µL (*8846*, long dsRNA 26%, siRNA 11%; *Bm_ago3*, long dsRNA 69%, siRNA 47%), which is consistent with the above results for other genes. We do not know whether the reduction in mRNA levels associated with these siRNAs is insufficient for the emergence of abnormal phenotypes or whether these genes are even involved in embryogenesis. Based on the results described above, we conclude that direct injection of siRNAs into eggs of *B. mori* effectively induces RNAi against genes of interest, by designing two pairs of siRNA.

### Importance of GC content around the target sequence

We also tested the efficiency of *odd*-siRNA-a and *odd*-siRNA-b individually. Injection of *odd*-siRNA-b had little effect on phenotype (5%), whereas *odd*-siRNA-a exhibited high efficiency (79%) (Table 1). According to the guideline presented in Fig. 1, *odd*-siRNA-b met criteria A to E, but not criterion F, as it was designed in a region with higher GC content (GC content of 58% in the 12 bp flanking sequences). In addition, we noted that the ineffective pair of *spz3*-siRNA-a and *spz3*-siRNA-b did not meet criterion F: the GC content in the 12 bp flanking sequences averaged 25% and 37%, respectively. These results suggest that criterion F is also important for the effective design of siRNAs and that regions with higher or lower GC content should be avoided as target positions for siRNAs.

### Dose-dependent effects at the mRNA level and on phenotype

To understand the dose-dependent effect of siRNAs, we selected *TH* as a target gene. *TH* is the first enzyme of the melanin synthesis pathway and plays an important role in pigmentation. The expression of *TH* increases as body pigmentation proceeds during the embryonic stage of *B. mori*. Recently, knockdown of *TH* in embryos using injection of long dsRNA yielded a pale brown coloration on the head and epidermis compared with black-colored wild-type animals [22]. Thus, we considered *TH* to be a good marker for evaluating the dose-dependent phenotypic effect of siRNAs.

**Figure 3:**
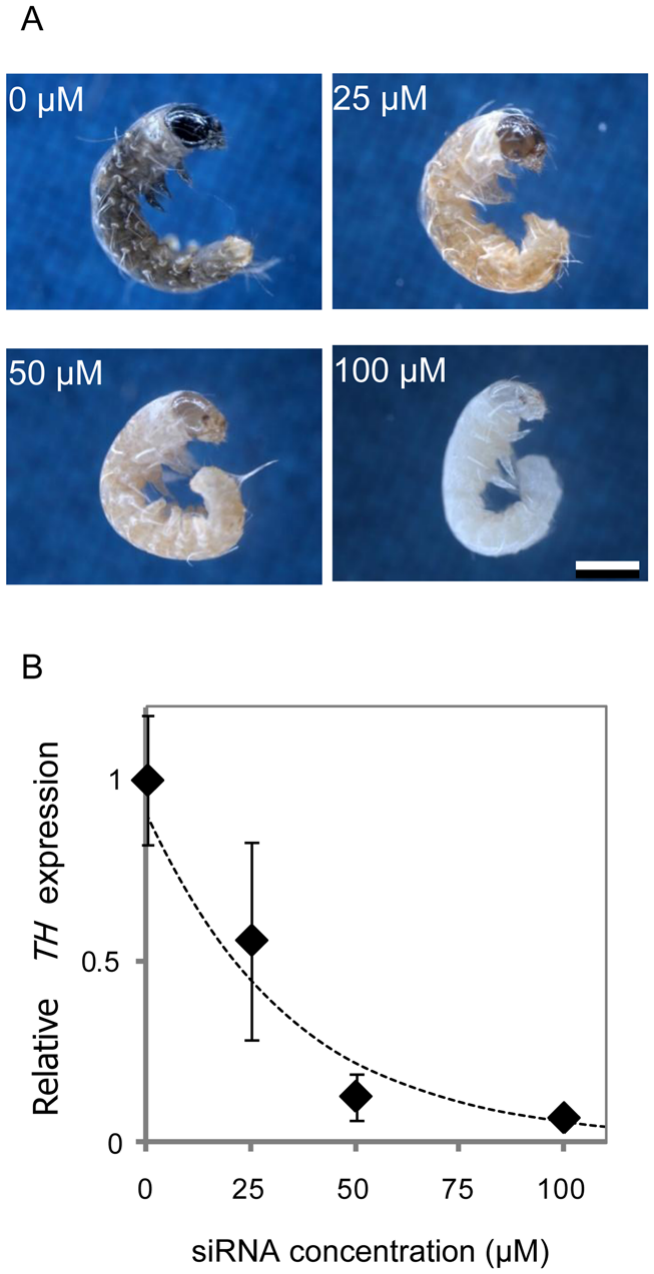
Dose-dependent effect of the *TH*-siRNA. (A) *TH*-siRNA effects on coloration in neonate larvae 9 days after oviposition. Dose-dependent effects were clearly observed from 0 to 100 µM. siRNAs were injected into eggs within 5 h of oviposition. Scale bar, 0.5 mm. (B) Quantification of *TH* mRNA levels 9 days after oviposition using real-time RT–PCR. The ribosomal protein L3 (*RpL3*) was used as an internal control. Error bar, S.D.; n = 3–4 individuals. The data were normalized and approximated by y = 0.9 e^−0.028 x^ (x, concentration of injected siRNA; y, relative amount of *TH*-mRNA; R = 0.9133). The median effective concentration was estimated at 21 µM.

We injected the *TH*-siRNA at different concentrations (0 µM, 25 µM, 50 µM, and 100 µM), and embryos were dissected for phenotypic observation 9 days after oviposition, just before hatching. The resulting phenotypes clearly exhibited a dose-dependent decrease in black pigmentation (Fig. 3 A). We also confirmed the dose-dependent reduction of the levels of the *TH* mRNA, which was correlated with the decrease in black pigmentation (Fig. 3 B). These results suggest that the effects of siRNAs are dependent on the quantity of RNA injected in the silkworm embryo, and that the enzymatic activity of *TH* is also dependent on the level of its mRNA.

The maximum concentration of 100 µM, which represents about 1.2 µg/µL of *TH*-siRNA in our experiment, caused complete repression of pigmentation, yielding a pale body color and translucent cuticle on the head and body (Fig. 3 A), A previous report showed that the injection of long dsRNA of *TH* at a concentration of 1 µg/µL caused “pale brown” individuals which left the pigmentation largely on the head and slightly on the body [22]. We also made and injected dsRNA of *TH* into the eggs and found that the dose-dependency and the same phenotype “pale brown” observed in a former report were confirmed (Fig. S2 A), However, even much higher concentration of long dsRNA (3.5 µg/µL, Fig. S2 A; 5.8 µg/µL, data not shown) could not induce “pale” phenotype as seen in the siRNA injection. We further tested that the pale phenotype obtained by injection of 100 µM of the *TH*-siRNA was not the result of a unspecific physiological response to dsRNA, because 250 µM of siRNAs targeting unrelated genes did not yield such a phenotype (data not shown). These results indicate that *TH*-siRNA induces stronger RNAi effect than long dsRNA of *TH*.

### Injection timing of siRNAs and duration of their effectiveness

Although it is known that the effect of injected dsRNAs is transient in the silkworm embryo, the suitable extent of the RNA injection or the duration of the effects of RNAi after injection are not certain at present; thus, we tried to answer these questions next. It is reported that strong expression of the *Ubx* mRNA is observed later than 60–80 h after oviposition [16]. As described above, *Ubx*-siRNA injection within 5 h of oviposition showed high efficiency (90%) of the characteristic phenotype (Table 1), suggesting that the effect of siRNA lasts for at least 60–80 h after injection. We further tested *Ubx*-siRNA injection 1 or 2 days after oviposition and found that the efficiency of the phenotypic change decreased drastically to 13% and 0%, respectively; conversely, the hatch rate increased to 41% and 50%, respectively. Cellularization and blastoderm formation in the silkworm embryo occur 9–14 h after oviposition [23], [24]; thus, the result described above indicates the possibility that the uptake of siRNA is disturbed by cellularization. However, we observed an efficiency of 14% for *Ubx*-siRNA injection 1 day after oviposition and some degree of effectiveness for the injection of *odd*-siRNA 2–3 days after oviposition (data not shown), suggesting that siRNAs can be incorporated even after cellularization, though their efficiency seems to decrease.

To ascertain the duration of the effectiveness of the *TH*-siRNA, we observed first instar larvae injected with 50 µM siRNA 5 h after oviposition and found that they were pale brown (Fig. 1 A). However, the color of these larvae changed to black at the second instar larval stage, which implies that the effect of injected siRNAs is lost before this stage. Reportedly, *TH* is expressed from 7 days to more than 10 days after oviposition [22], just after hatching, and in each of the larval molting stages. Therefore, the results described above indicate that siRNAs injected just after oviposition are stable and effective in eggs but are lost during the first instar larval stage.

### Effective concentration of siRNA

The amount of dsRNA used in RNAi experiments *in vivo* varies according to species and to the developmental stages of each animal, even within Lepidoptera [9]. In our experiments, 50 µM of siRNA were used mainly, and the median effective concentration of the *TH*-siRNA was 21 µM (Fig. 3 B). Kumar *et al.* reported that the acetylcholinesterase (*AChE*) gene was silenced by direct feeding (via an artificial diet) of less than 50 nM of siRNA to *Helicoverpa* larvae. The most prominent differences between this setting and our conditions may be the species or the different mechanisms, such as uptake, accumulation, and/or amplification of the dsRNAs. In the case of direct injection of long dsRNA into *B. mori* eggs, it was reported that 0.12–1.2 µg/µL (approximately 1–5 nL) was sufficient to induce the predictable embryogenesis phenotype [16], [17], [25] and to suppress the target mRNAs of *TH* to half the normal levels [22]. These data were consistent with our experiment (50 µM of siRNA; about 0.6 µg/µL), suggesting that the effective concentration of siRNA in eggs of *B. mori* depends on the weight concentration and not on the molar concentration.

### Conclusion

We demonstrated that various target genes can be silenced effectively by siRNA injection into silkworm eggs by satisfying the criteria A–F (see Fig. 1). The effectiveness of criteria A–D have been well studied in mammalian cells and in insect cells, and thought to relate to RISC formation by unwinding of the RNA duplex at the 3′ end of the siRNA [7]. By adding criteria E–H which were mainly identified empirically using mammalian cells [13]–[15, Japan Bio Services Co.,LTD.(JAPAN) unpublished data], we proposed the guideline to meet requirements for RNAi in *vivo* in *B. mori*, although it is not certain whether all of these criteria are indispensable for the effective design of siRNAs. We also clarified several essential conditions for the use of siRNA: injection timing, appropriate concentration, dose dependency, and duration of effectiveness. These may facilitate the comprehensive use of siRNA in reverse genetics not only in the silkworm but also potentially in a wide variety of insects.

In most cases we tested here, siRNAs could induce the stronger RNAi phenotype at more effective rates than long dsRNAs. This result indicates that siRNA is also useful for finding unknown effects which were somewhat difficult to be found by usual long dsRNA injection. Therefore, siRNAs can be used as complementary technique with higher effect of RNAi. As low-cost synthetic RNAs and genomic information for various insects are becoming available, this technique is useful for wide-scale screening of functional genes.

## Materials and Methods

### Experimental animals

*B. mori* strain N4 (non-diapause strain) was kindly gifted from Dr. Toru Shimada (University of Tokyo) and reared on an artificial diet (Silk Mate 2M, Nihon Nosan Kogyo. Co) at 25°C.

### Design and preparation of siRNA

Each 21-nucleotide siRNA sequence was designed based on a predicted gene in KAIKObase (China gene model) and EST database (CYBERGATE, http://150.26.71.213/cgi-bin/main_MX). The target positions were selected using siDirect ver. 2.0 (http://sidirect2.rnai.jp/) that automatically provides a list of potential candidates, and then we selected the appropriate sequences that satisfied the conditions described in Fig. 1 (see Results and Discussion). Usually two siRNAs were designed and synthesized for each target gene at different positions. They are mixed together and used in 50 µM in total. All sequences used in the experiments were listed in Table S1. The double-stranded siRNAs which were chemically synthesized and annealed were purchased from SIGMA Corp (Japan) and FASMAC Corp. (Japan). They were dissolved in the injection buffer (100 mM KOAc, 2 mM Mg(OAc)_2_, 30 mM Hepes-KOH, pH 7.4) and stored at −20°C until use.

### Injection of siRNA into eggs

The preparation of the eggs of *B. mori* was performed in a same way as described previously [26], [27]. Eggs of *B. mori* N4 stain were collected within 5 h after oviposition. The newly laid eggs were washed with tap water and left for 2–3 min. The floated eggs were transferred to glass slides and aligned in the same direction under a dissection microscopy. After eggs were dried for about 1 h at room temperature, they were bonded using an adhesive. The injection system was also a same as described previously, which consists of injector (Eppendorf, FemtoJet), manipulator (SURUGA SEIKI, M401), stepping motor controller (SURUGA SEIKI, M331) and dissection microscopy (Nikon, SMZ1500). First the small hole was made on the ventral side of the egg using the tungsten needle, subsequently the glass capillary was inserted to the hole and 1–5 nL of the siRNA solution was injected. The eggs were incubated in a Petri dish in a moist plastic box at 25°C until hatching.

### Real-Time RT-PCR

Total RNA was extracted using TRI reagent (SIGMA). After treatment with DNaseI (TaKaRa) to remove the genomic DNA followed by phenol-chloroform extraction and ethanol precipitation, precipitated RNAs were dissolved in water and were reverse transcribed with random primer (N_6_) using first-strand cDNA synthesis kit (GE health care). Real-time PCR was performed using power SYBR green PCR master mix on a StepOne system (Applied Biosystems) under the manufacture's recommended condition (denaturation at 95°C for 10 min, followed by 40 cycles of 95°C for 15 s, 60°C for 60 s). The house keeping gene ribosomal protein L3 (*RpL3*) was used as an internal control for normalization of sample loading. The sequences of the primer sets were listed in Table S2.

### Preparation of long dsRNAs

The PCR fragment of each gene was generated using the primers listed in Table S3 and cloned into pGEM T-easy plasmid (Promega). For the production of a template for *in vitro* transcription, PCR was performed using Ex taq polymerase (Takara) and two universal primers (T7-sense, 5′-taatacgactcactatagggagaccgcgggaattcgat-3′; and T7-anti, 5′-taatacgactcactatagggagagaattcactagtgat-3′) which contain T7 polymerase promoter sequence at their 5′ end. PCR products were purified using GenElute PCR clean-up kit (Sigma) for RNA synthesis using a MEGAscript T7 kit (Ambion). Sense and antisense transcripts were simultaneously synthesized using 0.6 µg PCR product. After DNase I treatment, RNA precipitated with LiCl was washed with 70% ethanol twice, dried, and dissolved in the injection buffer. The RNA solution was heated at 65°C for 30–60 min and cooled slowly to room temperature for annealing of dsRNA. The quality of dsRNA was examined by agarose gel electrophoresis. The concentration of each dsRNA was measured using Nano Drop ND-1000 spectrophotometer (Thermo fisher Scientific) and adjusted to final concentration of 2.4, 3.5 and/or 5.8 µg/µl.

## Supporting Information

Figure S1 **Other individuals with the characteristic phenotypes.** These phenotypes were obtained in a same condition as in Fig. 2. (A) The additional legs in A1 were observed when 50 µM of *Ubx*-siRNAs were injected into eggs within 5 h of oviposition (arrowhead). (B) Posterior segmentation was repressed when 50 µM of *wnt1*-siRNAs were injected into eggs within 5 h of oviposition.
(TIF)

Figure S2 **RNAi phenotype induced by long dsRNAs.** All long dsRNAs were injected under the same conditions as in the siRNA injection. (A) A “pale brown” phenotype of neonate larvae 9 days after oviposition, which was induced by long dsRNA of *TH*. A dose-dependent effect was observed as shown in the previous report [22] (left). A strong “pale” phenotype as seen after injection of *TH*-siRNA at 50–100 µM (0.6–1.2 µg/µL, Fig. 3A) was not observed even at higher concentration (3.5 µg/µL) of long dsRNA (right). Scale bar, 1 mm. (B) The characteristic phenotype of *Ubx* RNAi, which was induced by long dsRNA of *Ubx* (2.4 µg/µL) in 14% of injected embryos. The additional pair of leg-like protuberances in A1 segment was observed (black arrowhead), whereas the pair of spiracle of A1 remained (arrow). The detail of the leg-like protuberance with setae (white arrowhead) is shown in the right panel. This characteristic phenotype and the efficiency were consistent with a previous report [16] and with those of *Ubx*-siRNA injection (Fig. 2. A, B). In contrast, the lower concentration of *Ubx*-siRNA (0.6 µg/µL) induced the same phenotype at much higher efficiency (90%). (C) The characteristic phenotype of long dsRNA of *wnt1*. Two individuals with fused or truncated abdominal segments were shown, whereas no severe effects were observed on thoracic segments (arrowheads). Stronger phenotype was observed by the injection of *wnt1*-siRNA (Fig. 2 C, D) at the higher rate (long dsRNA, 9%; siRNA, 97%). (D) The effects of long dsRNA of *odd*. Some segmentation defects were observed similar to that of *odd*-siRNA injection (Fig. 2 E, F), but the effect was much lower; it was restricted to the thoracic segment (black arrowheads) but no abnormality was detected in abdominal segments (white arrowheads).
(TIF)

Table S1 **All sequences of the siRNAs.**
(PPT)

Table S2 **Primer list for real-time RT-PCR.**
(PPT)

Table S3 **Primer list for the long dsRNAs.**
(PPT)
