# Supplementary figures and images for: siRNAs Induce Efficient RNAi Response in Bombyx mori Embryos
Source: PLoS One. 2011 Sep 30;6(9):e25469. doi: 10.1371/journal.pone.0025469 (PMC3184131; doi:10.1371/journal.pone.0025469)

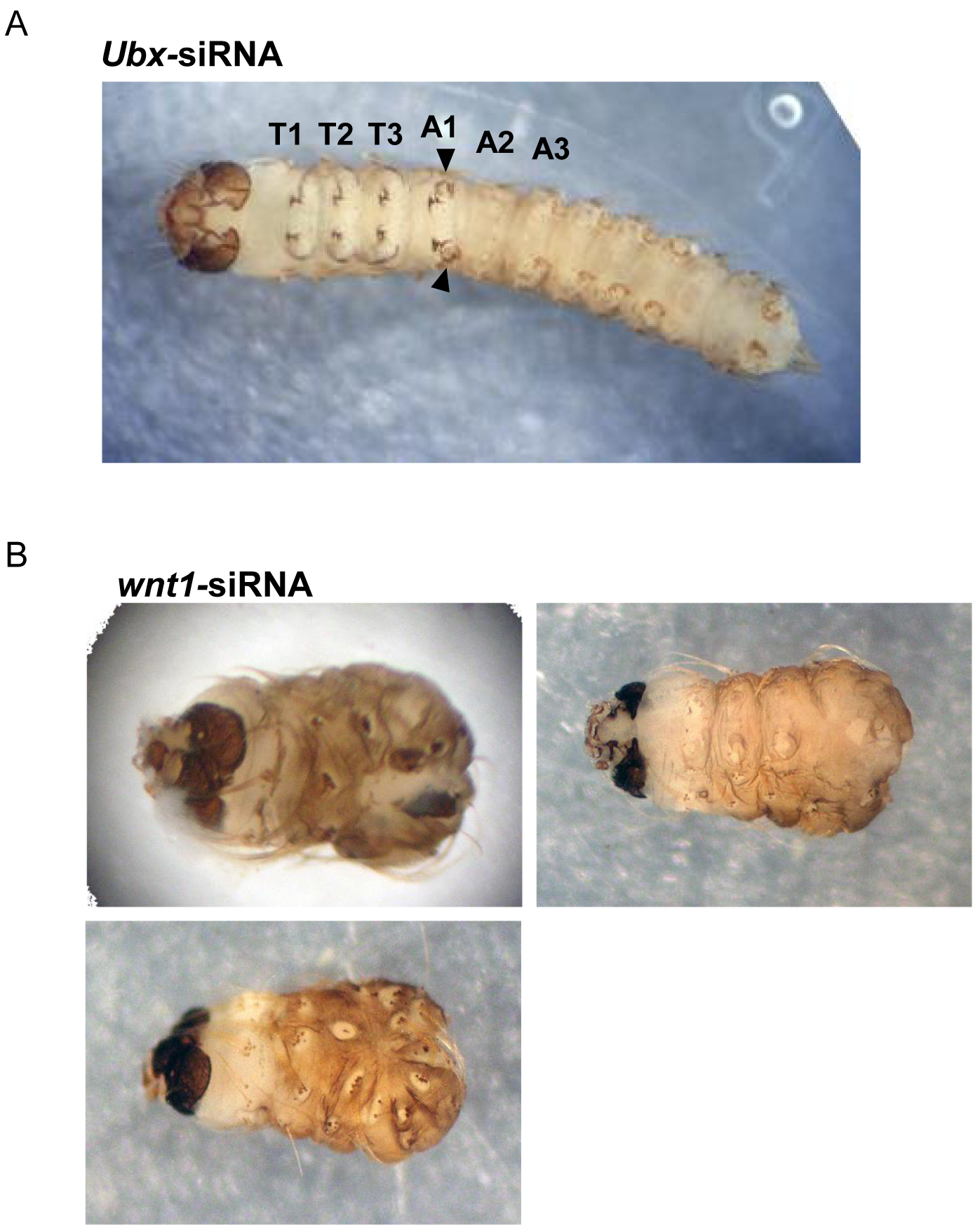

Supplement: Figure S1 — Other individuals with the characteristic phenotypes. These phenotypes were obtained in a same condition as in Fig. 2. (A) The additional legs in A1 were observed when 50 µM of Ubx-siRNAs were injected into eggs within 5 h of oviposition (arrowhead). (B) Posterior segmentation was repressed when 50 µM of wnt1-siRNAs were injected into eggs within 5 h of oviposition. (TIF) [file pone.0025469.s001.tif]

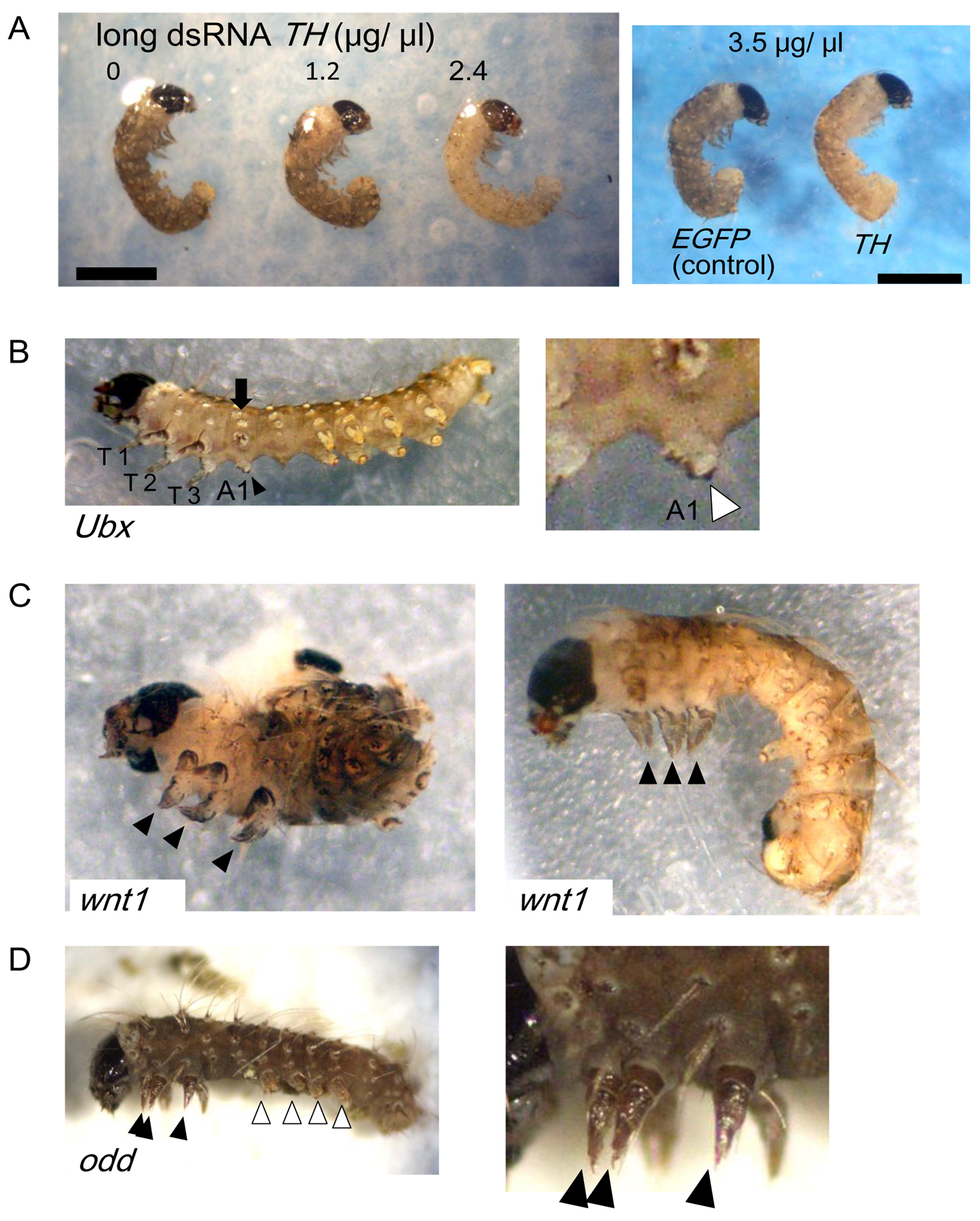

Supplement: Figure S2 — RNAi phenotype induced by long dsRNAs. All long dsRNAs were injected under the same conditions as in the siRNA injection. (A) A “pale brown” phenotype of neonate larvae 9 days after oviposition, which was induced by long dsRNA of TH. A dose-dependent effect was observed as shown in the previous report [22] (left). A strong “pale” phenotype as seen after injection of TH-siRNA at 50–100 µM (0.6–1.2 µg/µL, Fig. 3A) was not observed even at higher concentration (3.5 µg/µL) of long dsRNA (right). Scale bar, 1 mm. (B) The characteristic phenotype of Ubx RNAi, which was induced by long dsRNA of Ubx (2.4 µg/µL) in 14% of injected embryos. The additional pair of leg-like protuberances in A1 segment was observed (black arrowhead), whereas the pair of spiracle of A1 remained (arrow). The detail of the leg-like protuberance with setae (white arrowhead) is shown in the right panel. This characteristic phenotype and the efficiency were consistent with a previous report [16] and with those of Ubx-siRNA injection (Fig. 2. A, B). In contrast, the lower concentration of Ubx-siRNA (0.6 µg/µL) induced the same phenotype at much higher efficiency (90%). (C) The characteristic phenotype of long dsRNA of wnt1. Two individuals with fused or truncated abdominal segments were shown, whereas no severe effects were observed on thoracic segments (arrowheads). Stronger phenotype was observed by the injection of wnt1-siRNA (Fig. 2 C, D) at the higher rate (long dsRNA, 9%; siRNA, 97%). (D) The effects of long dsRNA of odd. Some segmentation defects were observed similar to that of odd-siRNA injection (Fig. 2 E, F), but the effect was much lower; it was restricted to the thoracic segment (black arrowheads) but no abnormality was detected in abdominal segments (white arrowheads). (TIF) [file pone.0025469.s002.tif]
