# Supplementary material for: siRNAs Induce Efficient RNAi Response in Bombyx mori Embryos
Source: PLoS One. 2011 Sep 30;6(9):e25469. doi: 10.1371/journal.pone.0025469 (PMC3184131; doi:10.1371/journal.pone.0025469)
Supplement: Table S1 — All sequences of the siRNAs. (PPT) [file pone.0025469.s003.ppt]

## Slide 1
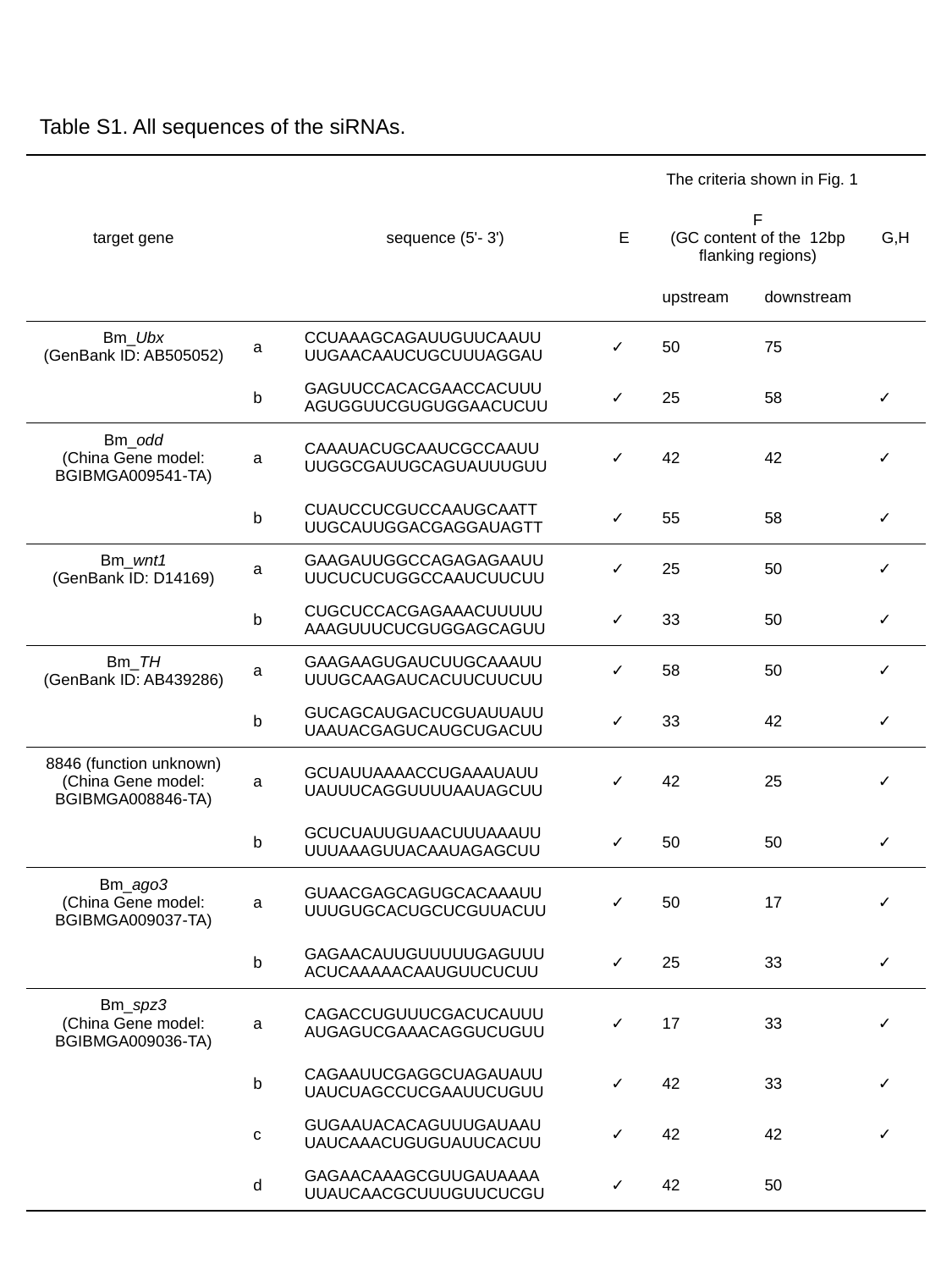

Table S1. All sequences of the siRNAs.
| | | | The criteria shown in Fig. 1 | | | |
| --- | --- | --- | --- | --- | --- | --- |
| target gene | | sequence (5'- 3') | E | F (GC content of the 12bp flanking regions) | | G,H |
| | | | | upstream | downstream | |
| Bm\_Ubx (GenBank ID: AB505052) | a | CCUAAAGCAGAUUGUUCAAUU UUGAACAAUCUGCUUUAGGAU | ✓ | 50 | 75 | |
| | b | GAGUUCCACACGAACCACUUU AGUGGUUCGUGUGGAACUCUU | ✓ | 25 | 58 | ✓ |
| Bm\_odd (China Gene model: BGIBMGA009541-TA) | a | CAAAUACUGCAAUCGCCAAUU UUGGCGAUUGCAGUAUUUGUU | ✓ | 42 | 42 | ✓ |
| | b | CUAUCCUCGUCCAAUGCAATT UUGCAUUGGACGAGGAUAGTT | ✓ | 55 | 58 | ✓ |
| Bm\_wnt1 (GenBank ID: D14169) | a | GAAGAUUGGCCAGAGAGAAUU UUCUCUCUGGCCAAUCUUCUU | ✓ | 25 | 50 | ✓ |
| | b | CUGCUCCACGAGAAACUUUUU AAAGUUUCUCGUGGAGCAGUU | ✓ | 33 | 50 | ✓ |
| Bm\_TH (GenBank ID: AB439286) | a | GAAGAAGUGAUCUUGCAAAUU UUUGCAAGAUCACUUCUUCUU | ✓ | 58 | 50 | ✓ |
| | b | GUCAGCAUGACUCGUAUUAUU UAAUACGAGUCAUGCUGACUU | ✓ | 33 | 42 | ✓ |
| 8846 (function unknown) (China Gene model: BGIBMGA008846-TA) | a | GCUAUUAAAACCUGAAAUAUU UAUUUCAGGUUUUAAUAGCUU | ✓ | 42 | 25 | ✓ |
| | b | GCUCUAUUGUAACUUUAAAUU UUUAAAGUUACAAUAGAGCUU | ✓ | 50 | 50 | ✓ |
| Bm\_ago3 (China Gene model: BGIBMGA009037-TA) | a | GUAACGAGCAGUGCACAAAUU UUUGUGCACUGCUCGUUACUU | ✓ | 50 | 17 | ✓ |
| | b | GAGAACAUUGUUUUUGAGUUU ACUCAAAAACAAUGUUCUCUU | ✓ | 25 | 33 | ✓ |
| Bm\_spz3 (China Gene model: BGIBMGA009036-TA) | a | CAGACCUGUUUCGACUCAUUU AUGAGUCGAAACAGGUCUGUU | ✓ | 17 | 33 | ✓ |
| | b | CAGAAUUCGAGGCUAGAUAUU UAUCUAGCCUCGAAUUCUGUU | ✓ | 42 | 33 | ✓ |
| | c | GUGAAUACACAGUUUGAUAAU UAUCAAACUGUGUAUUCACUU | ✓ | 42 | 42 | ✓ |
| | d | GAGAACAAAGCGUUGAUAAAA UUAUCAACGCUUUGUUCUCGU | ✓ | 42 | 50 | |
