# Supplementary material for: siRNAs Induce Efficient RNAi Response in Bombyx mori Embryos
Source: PLoS One. 2011 Sep 30;6(9):e25469. doi: 10.1371/journal.pone.0025469 (PMC3184131; doi:10.1371/journal.pone.0025469)
Supplement: Table S2 — Primer list for real-time RT-PCR. (PPT) [file pone.0025469.s004.ppt]

## Slide 1
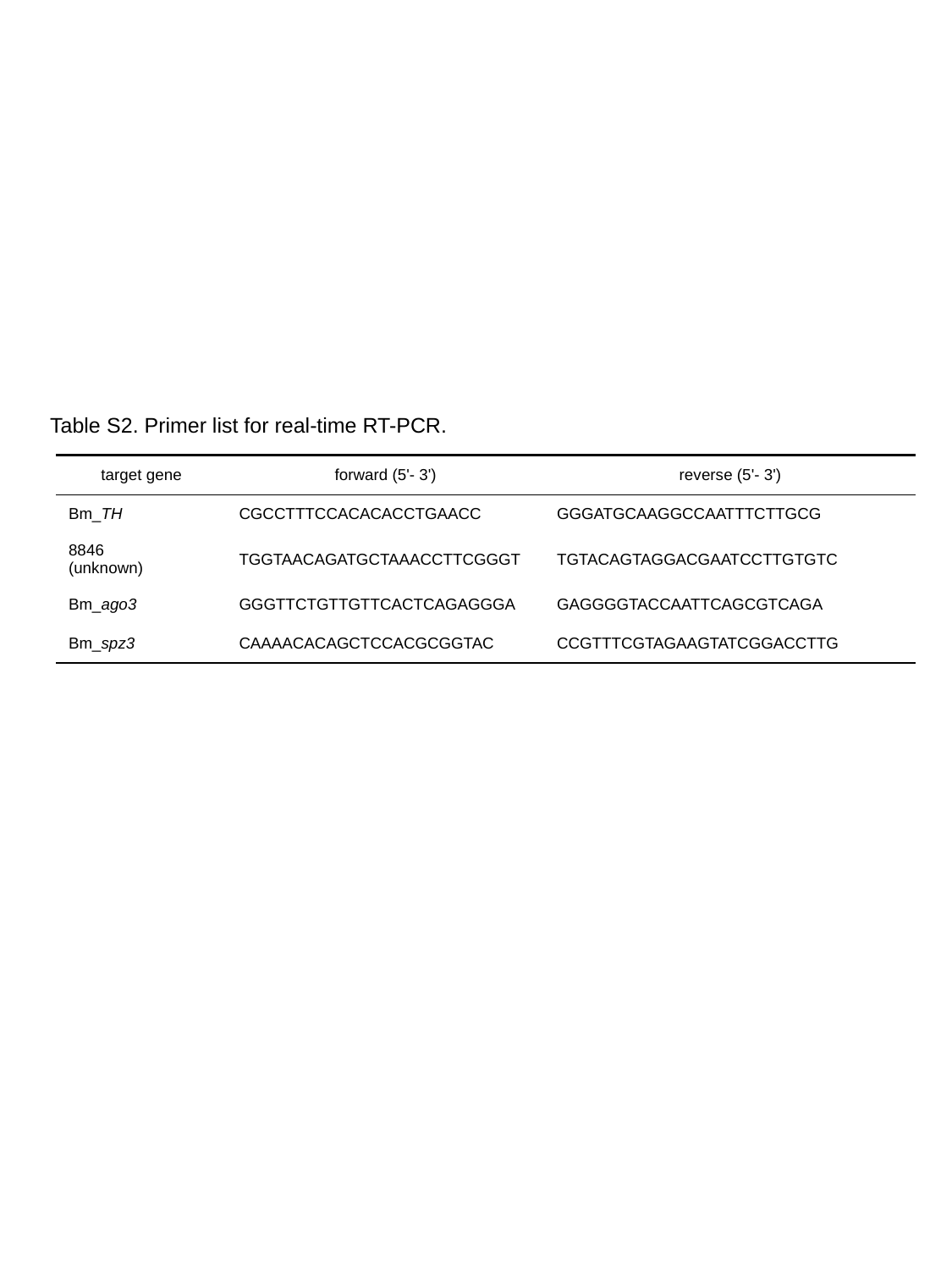

Table S2. Primer list for real-time RT-PCR.
| target gene | forward (5'- 3') | reverse (5'- 3') |
| --- | --- | --- |
| Bm\_TH | CGCCTTTCCACACACCTGAACC | GGGATGCAAGGCCAATTTCTTGCG |
| 8846 (unknown) | TGGTAACAGATGCTAAACCTTCGGGT | TGTACAGTAGGACGAATCCTTGTGTC |
| Bm\_ago3 | GGGTTCTGTTGTTCACTCAGAGGGA | GAGGGGTACCAATTCAGCGTCAGA |
| Bm\_spz3 | CAAAACACAGCTCCACGCGGTAC | CCGTTTCGTAGAAGTATCGGACCTTG |
