# Supplementary material for: siRNAs Induce Efficient RNAi Response in Bombyx mori Embryos
Source: PLoS One. 2011 Sep 30;6(9):e25469. doi: 10.1371/journal.pone.0025469 (PMC3184131; doi:10.1371/journal.pone.0025469)
Supplement: Table S3 — Primer list for the long dsRNAs. (PPT) [file pone.0025469.s005.ppt]

## Slide 1
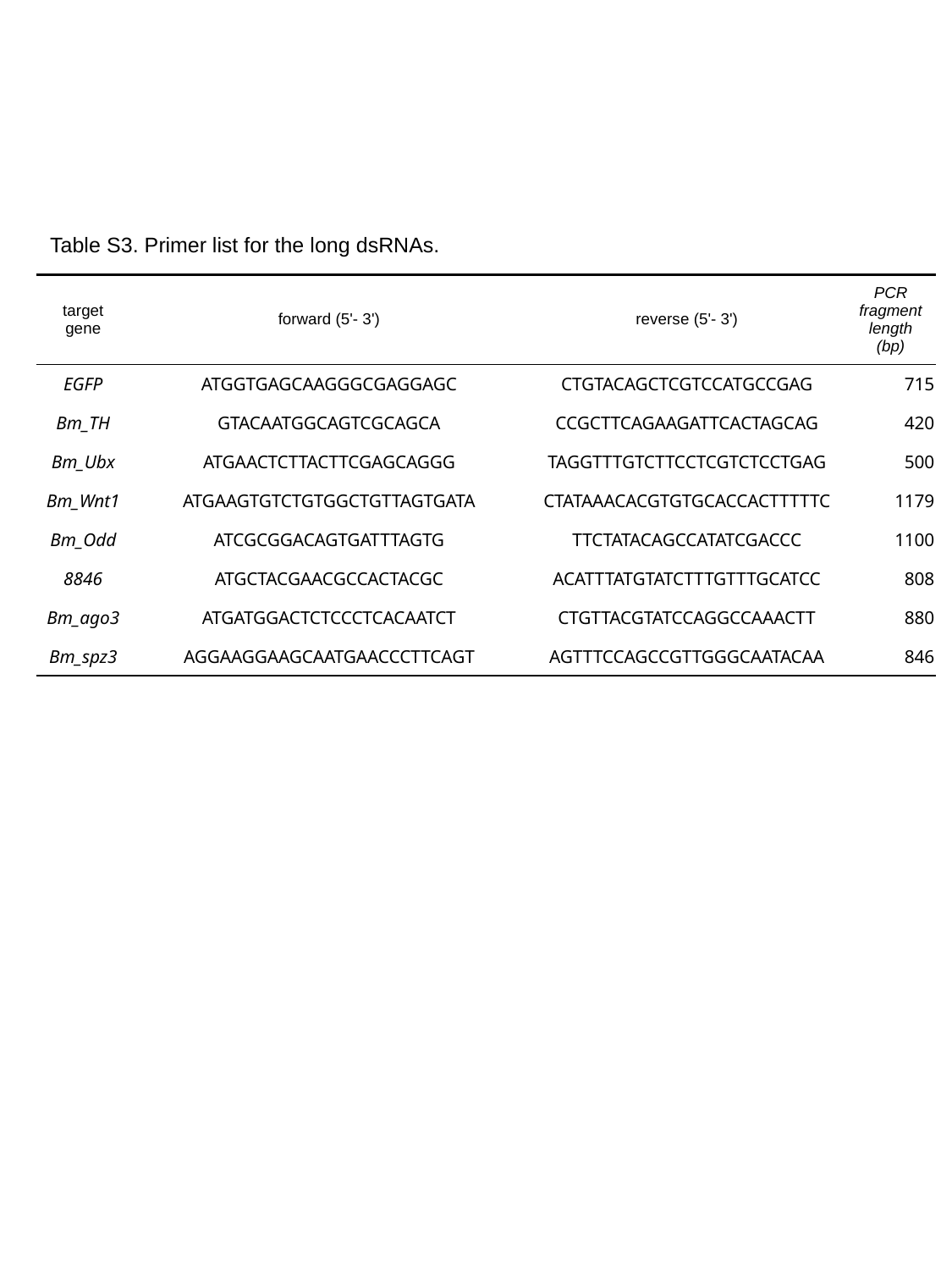

Table S3. Primer list for the long dsRNAs.
| target gene | forward (5'- 3') | reverse (5'- 3') | PCR fragment length (bp) |
| --- | --- | --- | --- |
| EGFP | ATGGTGAGCAAGGGCGAGGAGC | CTGTACAGCTCGTCCATGCCGAG | 715 |
| Bm\_TH | GTACAATGGCAGTCGCAGCA | CCGCTTCAGAAGATTCACTAGCAG | 420 |
| Bm\_Ubx | ATGAACTCTTACTTCGAGCAGGG | TAGGTTTGTCTTCCTCGTCTCCTGAG | 500 |
| Bm\_Wnt1 | ATGAAGTGTCTGTGGCTGTTAGTGATA | CTATAAACACGTGTGCACCACTTTTTC | 1179 |
| Bm\_Odd | ATCGCGGACAGTGATTTAGTG | TTCTATACAGCCATATCGACCC | 1100 |
| 8846 | ATGCTACGAACGCCACTACGC | ACATTTATGTATCTTTGTTTGCATCC | 808 |
| Bm\_ago3 | ATGATGGACTCTCCCTCACAATCT | CTGTTACGTATCCAGGCCAAACTT | 880 |
| Bm\_spz3 | AGGAAGGAAGCAATGAACCCTTCAGT | AGTTTCCAGCCGTTGGGCAATACAA | 846 |
